# Measurement of serum procalcitonin levels for the early diagnosis of spontaneous bacterial peritonitis in patients with decompensated liver cirrhosis

**DOI:** 10.1186/s12879-015-0776-4

**Published:** 2015-02-13

**Authors:** Zhao-Hua Cai, Chun-Lei Fan, Jun-Fu Zheng, Xin Zhang, Wen-Min Zhao, Bing Li, Lei Li, Pei-Ling Dong, Hui-Guo Ding

**Affiliations:** Department of Gastroenterology and Hepatology, Beijing You’an Hospital affiliated to Capital Medical University, Beijing, Fengtai District 100069 China; Department of Internal Medicine, The Second Hospital Beijing, Beijing, Xicheng District 100031 China

**Keywords:** Cirrhosis, Infection, Spontaneous bacterial peritonitis, Procalcitonin, Early diagnosis

## Abstract

**Background:**

It is difficult to diagnose spontaneous bacterial peritonitis (SBP) early in decompensated liver cirrhotic ascites patients (DCPs). The aim of the study was to measure serum procalcitonin (PCT) levels and peripheral blood leukocyte/platelet (WBC/PLT) ratios to obtain an early diagnostic indication of SBP in DCPs.

**Methods:**

Our cohort of 129 patients included 112 DCPs (94 of whom had infections) and 17 cases with compensated cirrhosis as controls. Bacterial cultures, ascitic fluid (AF) leukocyte and peripheral WBC/PLT counts, and serum PCT measurements at admission were carried out prior to the use of antibiotics. Receiver operating characteristic (ROC) curves were generated to test the accuracies and cut-off values for different inflammatory markers.

**Results:**

Among the 94 infected patients, 66 tested positive by bacterial culture, for which the positivity of blood, ascites and other secretions were 25.8%, 30.3% and 43.9%, respectively. Lung infection, SBP and unknown sites of infection accounted for 8.5%, 64.9% and 26.6% of the cases, respectively. Serum PCT levels (3.02 ± 3.30 ng/mL) in DCPs with infections were significantly higher than those in control patients (0.15 ± 0.08 ng/mL); p < 0.05. We used PCT ≥0.5 ng/mL as a cut-off value to diagnose infections, for which the sensitivity and specificity was 92.5% and 77.1%. The area under the curve (AUC) was 0.89 (95% confidence interval: 0.84–0.91). The sensitivity and specificity were 62.8% and 94.2% for the diagnosis of infections, and were 68.8% and 94.2% for the diagnosis of SBP in DCPs when PCT ≥2 ng/mL was used as a cut-off value. For the combined PCT and WBC/PLT measurements, the sensitivity was 76.8% and 83.6% for the diagnosis of infections or SBP in DCPs, respectively.

**Conclusion:**

Serum PCT levels alone or in combination with WBC/PLT measurements seem to provide a satisfactory early diagnostic biomarker in DCPs with infections, especially for patients with SBP.

## Background

One of the most common and serious complications in decompensated cirrhotic patients (DCPs) is bacterial infection [1-3]. Bacterial infections are known to be important causes of high mortality and morbidity in such patients, although antibiotics have been developed in recent decades [4]. Therefore, it has been suggested that the occurrence of bacterial infections should be considered as a further prognostic stage to define critical cirrhosis, which is associated with >40% increased mortality and longer hospital stays [2,5]. The most common infections in DCPs are cases of spontaneous bacterial peritonitis (SBP), which account for 40%–70% of cases, followed by urinary tract infections, pneumonia and cellulitis [3,6]. The early diagnosis of infections can improve the prognoses of patients [7]. However, it is difficult to diagnose SBP early in DCPs with ascites because the clinical manifestations and ascitic biochemical characteristics are often inconsistent [3-6]. Current guidelines state that culture positivity for a pathogen in ascitic fluid (AF) is the gold standard for the diagnosis of SBP. However, ascites culture has been negative in about 60% of patients with clinical manifestations suggestive of SBP and increased ascites neutrophil count [8,9]. Therefore, new studies of early diagnosis, prevention and treatment are needed to improve clinical outcomes. Many studies have shown that serum procalcitonin (PCT) is a sensitive biomarker that can be used to monitor bacterial infections, and measurements of PCT levels may guide the clinical use of antibiotics [10]. However, the diagnostic value of serum PCT levels in DCPs with infections [11], especially SBP, remains unclear, several studies provide conflicting results [12-16]. Therefore, this study aimed to determine the diagnostic value of serum PCT levels alone, or in combination with the peripheral blood leukocyte and platelet count (WBC/PLT) measurements in DCPs with bacterial infections and/or SBP.

## Methods

### Design

This was a retrospective cross-sectional case-controlled study to determine the early diagnostic value of serum PCT levels alone, or in combination with WBC/PLT measurements in DCPs with SBP.

### Patients

A cohort of 129 patients included 112 DCPs and 17 age- and sex-matched compensated cirrhosis controls who were enrolled for retrospective studies at the Department of Gastroenterology and Hepatology in Beijing You’an Hospital affiliated to Capital Medical University between January 2011 and June 2013 (Table 1). All subjects met the following criteria: (1) decompensated liver cirrhosis with ascites and/or other complications were confirmed by medical history, liver function assessments and B ultrasonography (LOGIQ9, General Electric, Fairfield, USA) or computerized tomography (CT; GE HISPEED DXI, General Electric) examinations were proven cirrhosis; (2) the compensated cirrhosis was histologically diagnosed or peripheral PLT was <100 × 10^9^/L and liver elastography stiffness was >12.5 kPa by FibroScan®502 (ECHOSENS, French); (3) the pathogen cultures, serum PCT measurements, blood and AF biochemistry tests and peripheral WBC/PLT counts were conducted before the use of antibiotics at admission; and (4) the patients did not exhibit liver failure, liver cancer or fungal infection or show serious heart, lung, or brain insufficiency, or have a mental illness.

**Table 1:** **Clinical characteristics of the enrolled patients**

		**DCPs with infections N = 94**	**DCPs without infections N = 18**	**Control N = 17**
Age (M ± SD)		59.84 ± 11.68	56.44 ± 12.4	54.05 ± 10.24
Gender (%)	Male	58 (61.7)	13 (72.2)	11 (64.7)
	Female	36 (38.3)	5 (27.8)	6 (35.3)
Etiology of cirrhosis (%)	Hepatitis B	52 (55.3)	13 (72.2)	13 (76.5)
	Hepatitis C	2 (2.1)	0 (0)	0 (0)
	Alcohol	21 (22.3)	5 (27.8)	4 (23.5)
	Others	19 (20.3)	0 (0)	0 (0)
Child–Pugh classification (%)	A	0 (0)	0 (0)	17 (100)
	B	5 (5.3)	7 (38.9)	0 (0)
	C	89 (94.7)	11 (61.1)	0 (0)
Number of complications (%)*	≥2	66 (70.2)	8 (44.4)	0 (0)
	<2	28 (29.8)	10 (55.6)	0 (0)

*Complications were included ascites or and esophageal variceal bleeding, hepatic encephalopathy or hepatorenal syndrome.

The etiology of cirrhosis was determined in cirrhotic patients by assessing hepatitis B and C status, alcoholism, cryptogenic disease and autoimmune cirrhosis from the medical record of each patient. The severity of cirrhosis was classified based on the Child–Pugh criteria, while all of the cirrhotic patients were evaluated for the presence of hepatocellular carcinoma using B ultrasonography and CT.

The study was approved by the Clinical Research and Ethics Committee of Capital Medical University, Beijing You’an Hospital.

### Paracentesis and AF culture

Diagnostic paracentesis was carried out at the bedside using a sterile method with a 22-G needle attached to a 20-cc syringe after local anesthesia with lidocaine. AF was immediately drawn from the peritoneal fluid after the sterile needle was attached to a syringe for paracentesis. Then, aspirated AF was collected into ethylenediaminetetraacetic acid tubes and analyzed for biochemistry and leukocyte counts within 3 h. We collected 10 mL AF from patients and cultured it in aerobic blood culture bottles (Becton Dickinson, Franklin Lakes, NJ, USA). These bottles were then placed into an automated Bactec 9120 3D culture system (Becton Dickinson) for 48–72 h. Bacterial identification and antimicrobial susceptibility testing were carried out using standard procedures with a Phoenix100 automated bacterial identification system.

### Bacterial culture of other samples

We inoculated 10 mL venous blood, urine or respiratory secretion samples taken from patients before the administration of antibiotics in blood culture bottles using the same methods as described above for the ascites bacterial culture.

#### Diagnostic criteria of infections

Bacterial infections in DCPs met sepsis and/or SBP criteria. Sepsis was determined by the following criteria: (1) fever (T >38°C); (2) peripheral WBC >10 × 10^9^/L; and (3) unexplained circulatory or renal failure and systolic blood pressure <90 mmHg after fluid resuscitation. Pneumonia was confirmed by lung X-ray and CT examination. Urinary tract infections were confirmed by positive urine bacterial cultures.

### Diagnostic criteria of SBP

SBP was determined based on one of the following criteria, as revised from available guidelines [8,9]: (1) abdominal pain and/or fever (T >37.5°C), and/or abdominal and rebound tenderness (excluding secondary peritonitis); and (2) AF leukocytes counts ≥250/mm^3^ and/or bacterial culture positivity.

### Serum PCT measurements

Serum PCT levels were measured using enzyme-linked fluorescence analysis (ELFA) with a detection limit of 0.1 ng/mL using an automated enzyme-linked fluorescence quantitative analyzer (VIDAS, Merieux, France).

### Assessments of blood biochemistry

Data from blood biochemistry tests were obtained from the medical records of each patient. These data included peripheral WBC/PLT counts (determined using a MEK 6318 K automatic blood cell analyzer, Nihon Kokden, Tokyo, Japan) and parameters from liver and renal biochemical profiles, such as alanine aminotransferase, aspartate aminotransferase, total bilirubin, albumin, blood urea nitrogen, and creatinine levels (as measured using an automatic biochemical analyzer, Olympus AU640, Tokyo, Japan).

### Statistical analyses

Qualitative data were expressed as n (%) and quantitative variables were expressed as the mean ± standard deviation (M ± SD). The Mann–Whitney *U* test was used to compare non-normally distributed quantitative data, and Pearson’s *χ*^2^ test was used to evaluate qualitative data. Statistical tests were performed using computer software (SPSS16.0, SPSS Inc. Chicago, IL, USA). The receiver operating characteristic (ROC) curves were generated to test the accuracies and cut-off values for different inflammatory markers. The optimal cutoff value of WBC/PLT was determined by calculating the point on the ROC curve with the maximum Youden index (sensitivity-[1-specificity]). While the cutoff values of WBC and PCT were determined according to the standard widely accepted in clinic [12-18]. The area under the curve (AUC) and 95% confidence interval (CI) were assessed where appropriate. A level for statistical significance was set at p < 0.05.

## Results

### Patient demographics

A total 94 of 112 DCPs had confirmed bacterial infections. These patients included 8 cases with lung infection (8.5%), 61 cases with SBP (64.9%) and 25 cases with an unknown site of infection (26.6%). A total of 66 of 94 infection cases showed bacterial culture positivity. The bacterial culture positivity rate of blood, AF and other secretion samples was 25.8% (17/66), 30.3% (20/66) and 43.9% (29/66), respectively. Among 61 DCPs with SBP, the bacterial culture positivity rate of AF was 32.8%. 70.2 percent (66/94) of DCPs with infections had at least two kinds of complications, including ascites, esophageal variceal bleeding, hepatic encephalopathy or hepatorenal syndrome.

### The diagnostic value of serum PCT levels and WBC/PLT ratios for detecting infections

**Figure 1:**
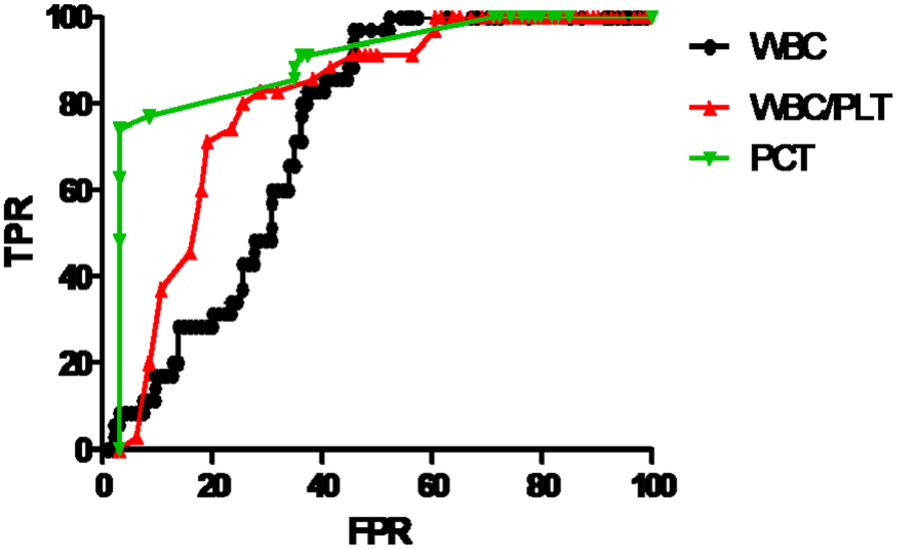
**Receiver operating characteristic (ROC) curves for PCT, WBC/PLT and WBC for diagnosis of infection in cirrhotic patients.** AUCs were 0.89 (95% CI: 0.84-0.91), 0.8 (95% CI: 0.72- 0.82 ) and 0.73 (95% CI: 0.64-0 .81) for PCT, WBC/PLT and WBC, respectively. The sensitivity ,specificity, PPV and NPV were 92.5%, 77.1%, 91.5%, and 79.4% at the cutoff of 0.5 ng/ml for PCT, 39.6%, 100%, 100%, and 38.1% at 0.25 for WBC/PLT, and 47.8%,100%,100% and 41.6% at 1.0 × 109/L for WBC. FPR=false positive rate (1- specificity), TPR= true positive rate (sensitivity), AUC= area under curve, CI = confidence interval, PPV=positive predictive value, NPV=negative predictive value.

Serum PCT levels (3.02 ± 3.30 ng/mL) in DCPs with infections were significantly higher than in control patients (0.15 ± 0.08 ng/mL); p < 0.05. There were no significant differences between serum PCT and WBC/PLT in cirrhotic patients with different sites of infection (Table 2). In peripheral blood, WBC ≥10 × 10^9^/L and WBC/PLT ≥0.25 was used as a cut-off to diagnose infections in DCPs, for which the AUC was 0.73 (95% CI: 0.64–0.81) and 0.8 (95% CI: 0.72–0.82), respectively (Figure 1). The cut-off value for serum PCT levels was 0.5 ng/mL for the diagnosis of infections in DCPs, for which the sensitivity and specificity were 92.5% and 77.1%, respectively. The AUC was 0.89 (95% CI: 0.84–0.91; Figure 1). The combination of serum PCT levels (cut-off: 2.0 ng/ml) and WBC/PLT ratios (≥0.25) can significantly improve sensitivity for the early diagnosis of infections in DCPs (Table 3).

**Table 2:** **Serum PCT levels, peripheral blood WBC and WBC/PLT ratios in the enrolled subjects**

**Group**	**PCT (ng/mL)**	**WBC** **(×10** ^**9**^ **/L)**	**WBC/PLT**
SBP (n = 61)	3.55 ± 3.53*	8.8 ± 1.1	0.23 ± 0.03*
Lung infection (n = 8)	3.2 ± 3.46*	6.7 ± 2.3	0.17 ± 0.06*
Unknown infections (n = 25)	2.24 ± 2.73*	5.1 ± 1.1	0.35 ± 0.07*
Control (n = 17)	0.15 ± 0.08	5.14 ± 1.04	0.05 ± 0.2

*Compared with control; p < 0.001.

**Table 3:** **The diagnostic efficacy of serum PCT levels combined with WBC/PLT ratios for infections in cirrhotic patients**

**Group**	**PCT**		**PCT + WBC/PLT**	
	**≥2 ng/mL**	**<2 ng/mL**	**Positive**	**Negative**
Infected (n = 94)	59	35	72	22
Non-infected (n = 35)	2	33	2	33
Sensitivity (%)	62.8		76.8	
Specificity (%)	94.2		94.2	
Positive predictive value (PPV, %)	96.7		97.8	
Negative predictive value (NPV, %)	48.5		60	

### The diagnostic value of serum PCT levels and WBC/PLT ratios for SBP

**Figure 2:**
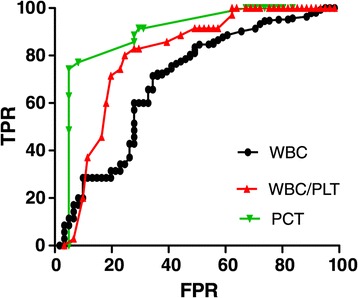
**Receiver operating characteristic (ROC) curves for PCT, WBC/PLT and WBC for diagnosis of SBP in cirrhotic patients.** AUCs were 0.89 (95% CI: 0.82-0.97), 0.79 (95% CI: 0.61-0 .87), and 0.73 (95% CI: 0.61-0.82), for PCT, WBC/PLT and WBC, respectively. The sensitivity ,specificity, PPV and NPV were 68.8%, 94.2%, 95.4%, and 63.5% at the cutoff of 2 ng/ml for PCT, 30.0%, 100%, 100%, and 47.9% at 0.25 for WBC/PLT, and 44.3%, 100%, 100%, and 50.7% at 1.0 × 109/L for WBC. FPR=false positive rate (1- specificity), TPR= true positive rate (sensitivity), AUC= area under curve, CI = confidence interval, PPV=positive predictive value, NPV=negative predictive value.

There were no significant differences in serum PCT levels or the WBC/PLT ratio between in AF culture-positive (2.92 ± 2.87 ng/mL, 0.25 ± 0.05) and culture-negative (2.88 ± 3.29 ng/mL, 0.23 ± 0.36) DCPs with SBP; p > 0.05. The diagnostic cut-off values for serum PCT levels (≥2.0 ng/mL), WBC (≥10 × 10^9^/L) and WBC/PLT (≥0.25) are shown in Figure 2, for which the AUC was 0.89 (95% CI: 0.82–0.97), 0.79 (95% CI: 0.61–0.87), and 0.73 (95% CI: 0.61–0.82), respectively. The combination of serum PCT levels and the WBC/PLT ratio could significantly improve sensitivity for the early diagnosis of SBP in DCPs (Table 4).

**Table 4:** **The diagnostic efficacy of serum PCT levels combined with WBC/PLT ratios for SBP in cirrhotic ascites patients**

**Group**	**PCT**		**PCT + WBC/PLT**	
	**≥2 ng/mL**	**<2 ng/mL**	**Positive**	**Negative**
SBP (n = 61)	42	19	51	10
Non-infected (n = 35)	2	33	2	33
Sensitivity (%)	68.8		83.6	
Specificity (%)	94.2		94.2	
Positive predictive value (PPV, %)	95.4		96.2	
Negative predictive value (NPV, %)	63.5		76.7	

## Discussion

Liver cirrhosis patients are very susceptible to bacterial infections because of acquired immune defects of both humoral and cell-mediated immunity and bacterial translocation [3,19]. Liver dysfunction is strongly associated with impaired defenses against bacteria, and with structural and functional alterations in the intestinal mucosa that lead to an increase in the permeability to bacteria and bacteria-derived products, which worsens over time and with disease progression. In end-stage liver disease patients, these events favor the translocation of bacteria, which increases susceptibility to infections, particularly SBP. The release of inflammatory mediators during infections leads to systemic, renal and hepatic hemodynamic impairment, which dramatically affects the prognosis, even after infections resolve [1,3]. More than 70% mortality in cirrhotic patients with drug-resistant infections was also reported [7,20]. It has been suggested that the early diagnosis of SBP, along with the prompt initiation of empirically based antibiotic therapy, have been considered to be crucial for the survival of a patient. Liver cirrhosis patients with sepsis caused by lung, urinary tract or skin infections were easy to diagnose clinically. To date, it remains a challenge for clinicians to arrive at an early diagnosis of SBP in cirrhotic patients with ascites because the early symptoms and signs are not obvious [5]. Biomarkers with high sensitivity and specificity for the diagnosis of SBP are lacking.

The diagnosis of SBP was based on AF polymorphonuclear (PMN) leukocyte counts >250/mm^3^ and positive bacterial cultures without any evidence of an external or intra-abdominal source of infection or malignancy according to all of the available guidelines [8,9]. However, on the one hand, despite the use of sensitive pathogen culture methods, ascites culture has been negative in as many as 60% of patients with clinical manifestations suggestive of SBP and with increased ascites neutrophil leukocyte counts [21,22]. On the other hand, performing an AF culture is time consuming and is not always an available option in an emergency. Therefore, the discovery of easy to use, rapid and reliable diagnostic biomarkers for SBP in DCPs was needed.

Levels of PCT, a propeptide of calcitonin with a long half-life of 25–30 h that is produced by peripheral blood mononuclear cells, significantly increases during the systemic response of an organism to an infection and has been hailed as a novel inflammatory biomarker for bacterial infections [23-25]. In more severe infections, higher serum PCT levels were observed. The specificity of PCT for the diagnosis of severe sepsis and septic shock is 100%, and was suggested to warn of the occurrence of multiple organ dysfunction [18,25]. Serum PCT measurements have been reported to be superior to C-reactive protein in discriminating infectious from other inflammatory diseases, such as acute pancreatitis, cardiogenic shock and acute transplant rejection [26,27]. Meanwhile, serum PCT can be rapidly and easily detected as early diagnostic biomarker for sepsis [23]. However, the utility of PCT as a marker for the early diagnosis of SBP has been reported limitedly, with conflicting results [12-16].

In this study, we found that serum PCT levels in cirrhotic patients with infections at admission were significantly higher than in non-infected patients and showed no relationship with the site of infection. Based on a serum PCT cut-off value of 0.5 ng/mL for the diagnosis of infections, the sensitivity and specificity were 92.5% and 77.1%, respectively. Based on our findings, the serum PCT cut-off values with the greatest specificity should routinely be 2 ng/mL. However, for a PCT cut-off value >2 ng/mL, the lower sensitivity may miss some cirrhotic patients with infections who should undergo early empirically based antibiotic treatment. Peripheral WBC is the most convenient diagnostic biomarker of infections, but it is often complicated by splenomegaly and hypersplenism in cirrhotic patients, so the WBC count was normally significantly lower. The study also found that in peripheral blood, WBC ≥10 × 10^9^/L and WBC/PLT ≥0.25 could be used for the diagnosis of cirrhotic patients with infections, for which the specificity was 100% but the sensitivity was lower (47.8% and 39.6%, respectively). Thus, based on our findings, the use of serum PCT levels combined with WBC/PLT ratios can significantly improve the sensitivity of the early diagnosis of liver cirrhosis with infections.

In this study, we also found that in DCP with SBP, serum PCT levels, WBC, and WBC/PLT ratios were not significantly different between ascites culture-positive and -negative patients. Serum PCT levels combined with WBC/PLT ratios was an early biomarker for the diagnosis of SBP, and the sensitivity and specificity were 83.6% and 94.2%, respectively. Based on rapid detection within 2 h, bedside availability and the non-invasiveness of the diagnostic examination, serum PCT levels appear to have many advantages over the traditional PMN count in AF for the early diagnosis of SBP, especially in the absence of another site of infection. It is strongly recommended for use in clinical practice for the early diagnosis of SBP in cirrhotic patients with ascites.

In conclusion, serum PCT levels along with WBC/PLT ratios can be used as diagnostic biomarkers of cirrhotic patients with infections. Serum PCT levels should be considered in combination with WBC/PLT ratios or AF leukocyte counts, which can significantly improve sensitivity for the early diagnosis of SBP. Without a doubt, a prospective, randomized multicenter studies are needed to confirm the early diagnostic value of serum PCT levels alone or in combination with WBC/PLT ratios or AF leukocyte counts and to guide empirically based antibiotic administration.

## Conclusions

From this study, we can conclude that serum PCT levels alone or in combination with WBC/PLT measurements seem to provide a satisfactory early diagnostic biomarker in DCPs with infections, especially for patients with SBP.
